# Contractions from Burns

**Published:** 1847-12

**Authors:** Bransby Cooper


*Contractions from Burns.* By Mr. Bransby Coopfr.—If suppuration take place in a burn, great care must be taken to prevent contractions during and after cicatrization, for both granulations and newly-formed skin have such a tendency to shrink, that all the precautions which can be taken will sometimes prove abortive, and dreadful distortions result.

I cannot agree with Mr. Earle in considering them always as a "source of blame to surgeons," but, on the contrary, believe that they are the effects of a condition of granulations and skin peculiar to burns, as indeed John Hunter has said:—"The contraction takes place in every point of the granulation, but principally from edge to edge, smaller and smaller, although there is little or no new skin formed, which now even has a similar power, assists the contraction of the granulations, and is generally more considerable than that of the granulations themselves; drawing the mouth of the wound together like a purse." These contractions produce the most frightful scars, and frequently interfere with the functions of important joints. I have often seen the chin drawn down upon the sternum, the lower jaw depressed so as to prevent the closing of the mouth, the saliva constantly flowing over the lips, deglutition obstructed, and all these inconveniences attended with frightful distortions of countenance. Permanent contractions of the limbs frequently result, tying the upper arm to the chest, after burns about the shoulders, so as entirely to prevent the performance of the motions of the upper extremity, rendering it often worse than useless, an actual burthen, and the inconvenience inducing the sufferer to seek any operation, even to the removal of the limb, for the cure of the deformity, rather than remain a permanent cripple. As to the operations proposed, and often performed, for the cure of the deformities arising from burns, I can say but little in their favour; and indeed so unsuccessful have they proved in my own practice, that I feel but little disposed to recommend a patient to submit to the painful dissections which are required for the removal of the cicatrices, and which so rarely afford the relief anticipated. Dupuytren, who does not speak very favourably of the result of operations on cicatrices after burns, recommends that they should never be attempted until months, or even years, after the formation of the cicatrix—not, in fact, until the "fræna and bands had become perfectly organized," when he considers they have no longer any tendency tc contract. An objection to this practice appears to me to present itself—viz., that the muscles have probably undergone a change to fit themselves to the new position of the limb; and that even bones themselves, if a joint be implicated in the contraction, may have also so accommodated themselves to the circumstances in which they are placed as to be incapable of being restored to their original position, even could this be effected.

The operation usually proposed for the removal of these deformities is the transverse division of the contracted cicatrices until the parts can be drawn and extended to their natural position, and there be retained by bandages and machinery competent to the purpose. In some cases this cannot be effected at once, but must be attempted by a slow and gradual extension, which should be constantly maintained during the granulating process. When these means are employed, the apparatus should be worn for many months after the wound is healed—indeed, until the new skin has become perfectly organized, or contraction will be certain to recur as soon as the extending force is removed. Should the granulations become too exuberant during the recovery, nitrate of silver should be freely employed to keep them down. Some surgeons have recommended that the whole extent of the cicatrized skin should be dissected off, and that the parts previously bound down should be restored as nearly as they can be to their natural position, that the edges of the healthy skin should be brought into as close an approximation as possible, and appropriate means employed to promote the healing of the wound. Modifications of this mode of treatment have been adopted by making a longitudinal incision through the healthy skin on each side of the wound, but at some distance from it, so as to enable you to bring the two portions of the skin, thus let loose as it were, in as close contact as possible. Some surgeons have even practised the Taliacotian plan of partially removing a portion of skin from the vicinity of the wound, and thus attempting to restore the burnt surface by applying it over the wound, and to unite it, by what is termed the plastic mode; but even under such circumstances, the contractions have returned with the same inveteracy as under the other modes of treatment.

I believe the great object is to prevent these contractions, during the progress of the healing of the wound, and the position of the patient is the principal preventive means, taking care that the mechanical methods are persevered in until the cicatrix is perfectly organized, and the new skin has lost all its tendency to contraction; but I acknowledge it is very difficult to say when that exact period has arrived.

About three years ago I had placed under my care a child of eight years old, who had been burnt about two years before in the front part of the neck; and in consequence of the contraction of the cicatrix, the head was drawn downwards toward the chest, the lower jaw and lip depressed, and the countenance much disfigured. I made an incision along the base of the lower jaw to the extent of the cicatrix, and dissecting the skin of the lip from the bone, restored it to its natural position. I then dissected off the whole of the cicatrix, divided the sternal attachment of the sterno-cleido mastoideus muscle on one side, and was then able to elevate the head; in which position I secured it by an apparatus, which was made for the purpose, to support the chin, and prevent subsequent retraction. The plan promised complete success for six or eight weeks, when the pressure of the chin upon the instrument, probably from contraction recurring, became so painful that its use was obliged to be discontinued. The result of this was, that, although the patient was relieved to a considerable degree, the deformity returned to a certain extent, and she left London permanently disfigured. One great advantage, however, was gained by this operation—viz., the lips were restored to perfect adaptation, and rendered capable of performing their natural functions. I have also divided cicatrices from burns in the hands producing contractions of the fingers, but with no better succcss. The protracted period necessary to the cure tires most persons out long before it is completed.—*Med. Gaz.*

